# Neuromuscular Control and Performance Differences Associated With Gender and Obesity in Fatiguing Tasks Performed by Older Adults

**DOI:** 10.3389/fphys.2018.00800

**Published:** 2018-07-03

**Authors:** Xu Duan, Joohyun Rhee, Ranjana K. Mehta, Divya Srinivasan

**Affiliations:** ^1^Department of Industrial and Systems Engineering, Virginia Tech, Blacksburg, VA, United States; ^2^Environmental and Occupational Health, Texas A&M University, College Station, TX, United States; ^3^Industrial and Systems Engineering, Texas A&M University, College Station, TX, United States

**Keywords:** motor variability, muscle activation, force fluctuation, intermittent contraction, knee extension

## Abstract

Obesity rates in the geriatric population have emerged as a serious health concern in recent decades. Yet, obesity-related differences in neuromuscular performance and motor control during fatiguing tasks, and how they are modified by gender, specifically among older adults, are still largely unexplored. The first aim of this study was to understand obesity and gender-related differences in endurance time among older adults. Motor variability has been linked with inter-individual differences in the rate of fatigue development, and as potentially revealing underlying mechanisms of neuromuscular control. Hence, the second and third aims of this study were to investigate to what extent motor variability at baseline could predict inter-individual differences in endurance time, and whether systematic obesity and gender differences exist in motor variability among older adults. Fifty-nine older adults (65 years or older) were recruited into four groups: obese male, obese female, non-obese male, and non-obese female. Participants performed submaximal intermittent isometric knee extensions until exhaustion. Knee extension force and muscle activation signals (surface electromyography) of a primary agonist muscle, the Vastus Lateralis (VL), were collected. Endurance time and metrics quantifying both the size and structure of variability were computed for the force and EMG signals, using coefficient of variation (within cycles and between cycles) and sample entropy measures. While group differences in endurance time were primarily associated with gender, adding individual motor variability measures as predictor variables explained significantly more variance in endurance time, thus highlighting the relevance of motor variability in understanding neuromotor control strategies. Males exhibited longer endurance times, higher EMG CV, lower EMG SaEn, lower force CV, and higher force SaEn than females. These findings are interpreted to indicate males as using a motor strategy involving better “distribution” of the neural efforts across synergists and antagonists to achieve better performance during the knee extension task. No obesity-related changes in endurance time were found. However, obese individuals exhibited a greater cycle-to-cycle variability in muscle activation, indicating a larger alteration in the recruitment of motor units across successive contractions and potentially increased neural costs, which may have contributed to comparable endurance time and performance as non-obese older adults.

## Introduction

Muscle fatigue, defined as the decline in muscular capacity to generate force, has been the focus of numerous investigations. Fatigue has been suggested to be related to impairment of neuromuscular performance, development of musculoskeletal disorders (MSDs), and occupational injuries (Takala, 2002; Gallagher and Schall, 2017). Muscle fatigue can be caused by many different factors, ranging from the accumulation of metabolites within muscle fibers to the generation of an inadequate motor command in the motor cortex (Enoka and Duchateau, 2008). Multiple individual factors such as gender, age, body mass index (BMI), pain status, health condition (e.g., previous injury history), experience, and task-specific factors including nature of task (static/dynamic) and muscle groups used, have been recognized as influencing fatigue-related performance declines in adults (Bemben et al., 1996; Madeleine and Madsen, 2009; Cavuoto and Nussbaum, 2013a; Hunter, 2014; Mehta and Cavuoto, 2015, 2017; Shortz and Mehta, 2017).

Aging is associated with several morphological and functional impairments in skeletal muscles that lead to a decrease in muscle force production capacity, which has consequently been associated with reduced physical function and independence in aging adults. Age-related changes in muscle fiber composition and reduction of active tissues have been linked to declining physiological capabilities such as reduced muscular strength and speed (Frontera et al., 1991; Hughes et al., 1999; Kent-Braun and Ng, 1999). Declining motor performance in older adults has been observed in several studies, especially in force-control tasks where older adults have been shown to be weaker and less steady (i.e., they exhibit greater fluctuations in force around a target force) than young adults (Enoka et al., 2003; Yoon et al., 2008). As increasing numbers of older adults are staying employed, and workers in the age group 55–64 have also been reported to suffer from the highest incidence rate of occupational injuries and illnesses, a better understanding and characterization of functional performance and associated neuromuscular control mechanisms among older adults is critical. This study specifically examines the performance of force-control tasks by older adults. The effects of two relevant and likely significant factors—gender and obesity—are considered, as elaborated below.

Obesity rates in the United States have dramatically risen in the last decade. The prevalence of obesity has recently been reported to be 37.7%, with a greater prevalence in women than men (35.0% men vs. 40.4% women) (Fryar et al., 2016). Several functional impairments have been found to be associated with obesity, e.g., endurance time has been shown to be significantly decreased for obese when compared to non-obese individuals in isometric and intermittent handgrip and shoulder flexion (Eksioglu, 2011; Cavuoto and Nussbaum, 2013a, 2014) and trunk exercises (Mehta and Cavuoto, 2017). Another study found that force fluctuation was significantly higher in the obese group compared to the healthy group in intermittent hand-grip exertions (Shortz and Mehta, 2013). Kankaanpää and colleagues found that women with high BMI fatigued faster than women with normal or low BMI in isometric back endurance tests (Kankaanpää et al., 1998). However, contrary results were observed in another study conducted by Cavuoto and colleagues, involving only young people aged below 30 years, as no significant differences in endurance time were observed between obese and non-obese groups in sustained isometric torso extension (Cavuoto and Nussbaum, 2013b), thus suggesting possible interactive influences of gender and age on the association between obesity and endurance.

Fat mass has been shown to increase with age, and is higher among later birth cohorts, peaking at about age 60–75 years (Rissanen et al., 1988; Drøyvold et al., 2006; Ding et al., 2007), whereas muscle mass and strength start to decline progressively around the age of 30 with a more accelerated loss after the age of 60 (Bassey, 1998; Rantanen et al., 1998; Frontera et al., 2000; Stenholm et al., 2008). Due to such physiological changes with aging, sarcopenic obesity (i.e., obesity and low muscle mass) may be more likely to occur in older adults, resulting in reduced body strength relative to their body size (Stenholm et al., 2008). This, in turn, may expose obese older adults to more risks of suffering from neuromuscular declines, and consequently being injured. Thus, the combined effect of obesity and aging is a critical neuromuscular risk factor that deserves to be studied in more detail.

Gender differences have been widely explored in the biomechanics, occupational, and motor control research communities in terms of performance and motor control changes with fatigue. Despite previous studies claiming gender differences in endurance, there were no differences in endurance or perceived fatigue when women and men were matched for strength (Hunter and Enoka, 2001; Hatzikotoulas et al., 2004), suggesting that earlier reported differences between men and women may probably be a strength effect, and not primarily related to gender. In contrast, gender differences were found in muscle contractile function and metabolism: the majority of force potentiation occurred very rapidly (i.e., after baseline strength testing) in males, whereas potentiation reached its maximum later, during an exercise protocol, in females; in an incremental isometric exercise, males exhibited a greater reliance on non-oxidative sources of ATP compared with females (Kent-Braun et al., 2002). Another recent study showed that although females and males did not differ in endurance time in a repetitive pointing task, there were gender differences in the relative contributions of the shoulder and elbow toward maintaining the same multi-joint movements with fatigue (Srinivasan et al., 2016). These results from healthy young individuals suggest that the underlying mechanisms of muscle response or motor control/coordination to fatigue may be different between genders, even though these may not manifest directly as difference in endurance or performance under fatiguing conditions. While this may indeed be the case among young and non-obese individuals, it is likely that such gender differences in motor control and muscle responses, when combined with obesity and old-age, may have stronger effects on performance among an older cohort of obese and non-obese individuals.

Overall, several studies have investigated functional impairments associated with obesity and gender (separately) in young adult groups. However, obesity-related differences in neuromuscular performance and motor control during fatiguing tasks among older adults, as well as how they are modified by gender, are still largely unexplored.

In understanding performance changes with fatigue, an increasing number of studies have recently emphasized the links between variations in movements, muscle activities, and coordination with fatigue development. This phenomenon, referred to as “motor variability,” is the natural variation in postures, movements and muscle activity observed to different extents in all tasks, even when an individual tries to achieve identical performance across repeats (Srinivasan and Mathiassen, 2012). Motor variability has been recognized to be ubiquitous, as it has been shown to occur even in highly controlled repetitive tasks (Fethke et al., 2007; Jackson et al., 2009). An interest in motor variability has emerged in occupational research due to its associations with pain/discomfort, fatigue and performance (Srinivasan and Mathiassen, 2012); in a clinical context focusing on pain, aging, diseases (van Emmerik and van Wegen, 2000; Heiderscheit et al., 2002), in rehabilitation (Field-Fote and Tepavac, 2002; Daly et al., 2007), and in sports biomechanics because motor variability is associated with performance and injury risk (Davids et al., 2003; Bartlett et al., 2007; Preatoni et al., 2010).

Fluctuations, or variabilities, have been demonstrated to exhibit a degree of order that can be attributed to the operation of an adaptive control system (Falla and Farina, 2007). More variability in motor strategies has been suggested to be representative of new motor solutions to look for ways to reduce fatigue-induced discomfort and deterioration in performance. Studies have shown that inter-individual differences in motor variability may be an important factor in determining individual differences in susceptibility to developing fatigue, pain, and musculoskeletal disorders caused by repetitive tasks: specifically in the context that higher motor variability may be related to stronger resistance to muscle fatigue and better adaptation to task demands (van Dieën et al., 1993; Mathiassen et al., 2003; Madeleine, 2010; Srinivasan and Mathiassen, 2012). Thus, motor variability may be a useful and relevant construct to understand the neuromuscular strategies employed by different individuals during the performance of fatiguing tasks.

Given the trends of increased aging and obesity in the population, and potential gender differences on neuromuscular performance, the first aim of this study was to determine obesity and gender differences in endurance time, among older adults performing intermittent knee extension endurance tasks until exhaustion. Due to the potential for motor variability to explain individual differences in motor strategies employed to preserve performance with fatigue, the second aim of this study was to determine whether motor variability at baseline could predict individual differences in endurance time of older adults. Finally, whether gender and obesity were systematically associated with group differences in motor variability and neuromuscular control were examined, using force and muscle activation variability as the dependent measures. Knee extension tasks were specifically investigated in this study since the knee is important for controlling balance and movement, and for performing a majority of occupational and daily living tasks. Also, in many cases, particularly for older adults, lower extremity muscle function is critical for accomplishing several basic tasks of functional mobility, such as transition from sitting to standing. Variability was quantified in the force and muscle activation (i.e., surface electromyography) signals using metrics to reflect: signal steadiness (coefficient of variation over the contraction period of each cycle of contraction), cycle-to-cycle variability (quantified as a between-cycle coefficient of variation, indicative of a variable motor-unit recruitment pattern across repeated cycles) and sample entropy (quantifying the complexity of the signal, indicative of the communication/coordination across neuromuscular compartments). Based on the evidence from prior literature, we expected that endurance time would be shorter in the obese group than the non-obese group, and the extent of obesity-related differences may be different across genders. We also expected that a higher cycle-to-cycle variability and sample entropy in muscle activations at baseline may be associated with prolonged endurance among individuals. Males and females, and obese and non-obese individuals were expected to systematically differ in variability of muscle activities, reflecting different neuromuscular control mechanisms, which may potentially be associated with gender and obesity differences in force fluctuation and endurance time.

## Methods

### Participants

Fifty-nine right-hand dominant older adults, 65 years or older, were recruited from the local community who formed four experimental groups: obese male (*n* = 13), obese female (*n* = 16), non-obese male (*n* = 15), and non-obese female (*n* = 15). Participants whose body mass index (BMI) were greater than 30 kg/m^2^ were considered to be obese and less than 25 kg/m^2^ were considered to be non-obese. The demographic data of the participants are shown in Table 1. All participants were self-reported to be sedentary to recreationally active individual without any musculoskeletal injuries or known disorders within the past year. The participants provided written informed consent before participation in the study, and the Institutional Review Boards at Texas A&M University and Virginia Tech approved the procedures.

**Table 1:** Demographic data.

**Group**	**Number**	**Age (years)**	**Stature (cm)**	**Body mass (kg)**	**BMI (kg/m^2^)**	**Knee extension strength (Nm)**	**Waist circumference (cm)**	**Average steps/day during previous 1 week**
Non-obese male	15	74 (6)	178 (7.8)	77 (8.6)	24.45 (1.22)	107.94 (30.59)	99.06 (4.94)	7,553 (2,368)
Non-obese female	15	72 (5)	164 (5.8)	61 (6.9)	22.7 (1.89)	72.35 (13.54)	88.37 (9.32)	7,726 (2,907)
Obese male	13	73 (7)	178 (8.3)	114 (16.8)	36.0 (3.4)	104.77 (45.7)	127.49 (10.36)	4,181 (1,744)
Obese female	16	72 (5)	160 (4.6)	98 (16.9)	37.9 (5.38)	62.04 (19.67)	118.82 (11.63)	4,833 (1,558)

*Note that all measures are presented as mean (SD)*.

### Procedures

Upon consent, participants were instrumented with activPAL™ physical activity monitor (Pal Technologies Ltd, Scotland, UK) on the right thigh using double-sided medical grade tape and a medical grade dressing patch, for one continuous week of measurement. Acceleration data were recorded at 20 Hz, and used to compute the number of steps per day (Tudor-Locke and Bassett, 2004). The average wear time across all participants was 6.84 days and 55 of 59 participants wore the sensor for all 7 days of monitoring.

Participants were then instrumented with relevant bio-instruments. The knee force exertions were measured using an isokinetic dynamometer (Humac NORM, Computer Sports Medicine, Stoughton, MA). Participants were seated upright with the dominant (right) hip and knee flexed to 90°. The epicondyles of their femur were aligned with the dynamometer's center-of-rotation and then locked with a dynamometer pad secured above the ankle, anterior to the tibia. The participant's upper body was secured firmly in the chair to minimize upper body motion caused by lower extremity muscle contraction. Three isometric maximum voluntary contractions (MVCs) in the posture described above, were measured prior to knee extension endurance test for each participant. Each subject was verbally encouraged to achieve maximal force. Two minutes of rest were provided in between each MVC trial to ensure adequate recovery. The greatest force achieved by the subject was taken as the MVC force. The target force level of 30% MVC was determined accordingly for the following experimental trials.

**Figure 1:**
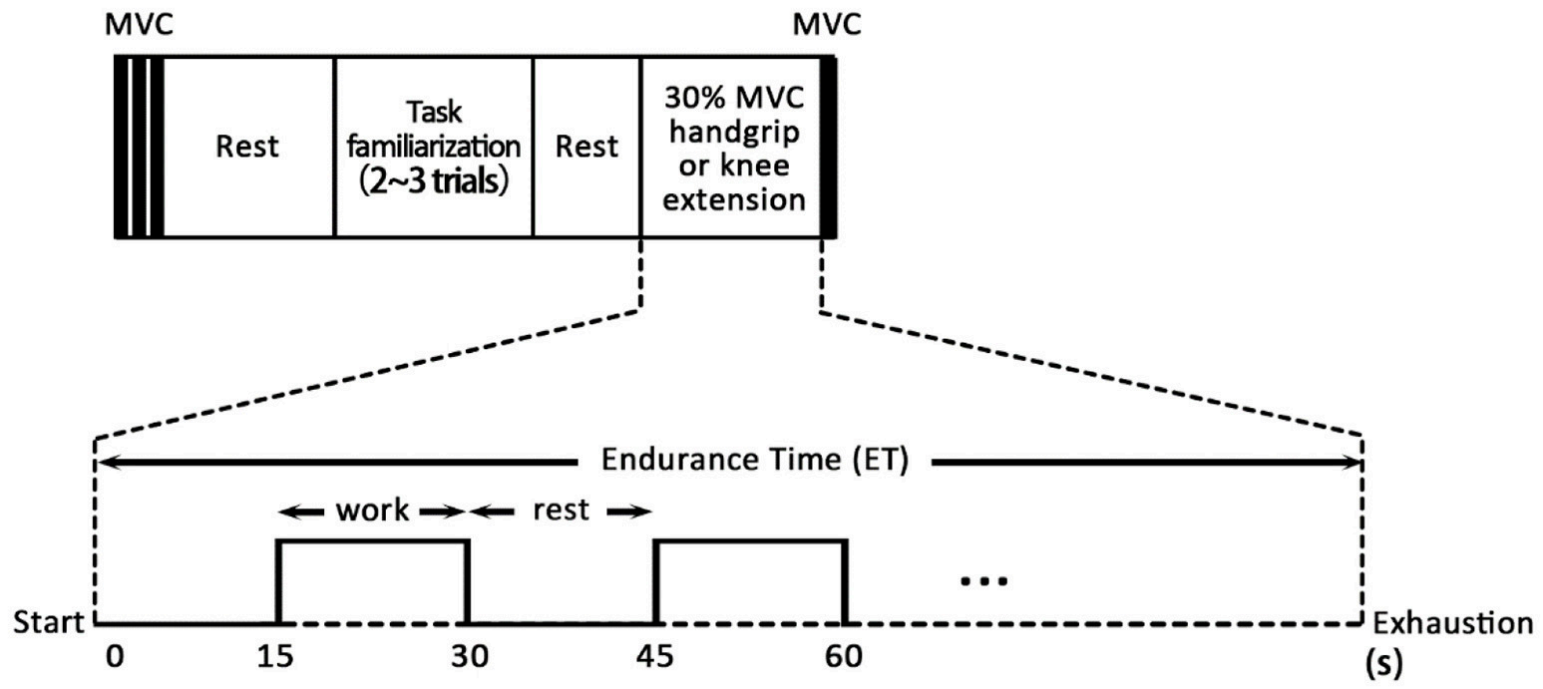
Experiment Protocol.

Following the MVC trials and prior to the actual experimental data collection, participants were provided with practice sessions to familiarize with the task. After familiarization and a period of adequate rest, participants performed intermittent isometric knee extension endurance test at 30% MVC until exhaustion. Each contraction lasted for 15 s with 15 s rest between each trial (Figure 1). Participants were asked to control their force generation against the target moment on the screen, which corresponded to a force of 30% MVC, as closely as possible based on real-time visual feedback provided. Participants continued performing the task until they were not able to maintain the 30% MVC force during the fifteen-second exertion period or until they reported as being unable to continue.

### Measurements

Knee extension electromyogram (EMG) signals were recorded at 1,000 Hz during the entire exercise session (Biopac Inc., Ca, USA, Biopac MP Systems). The skin above the muscle belly was cleaned using alcohol before placing the EMG electrodes. Muscle activities of the rectus femoris (RF) and vastus lateralis (VL) were recorded using surface EMG for the knee extension exercise. Knee extension force was recorded at 100 Hz (Humac Norm Isokinetic Dynamometer, Computer Sports Medicine, Stoughton, MA). The recorded signals were transmitted to a dedicated PC using an A/D converter.

### Data analysis

For physical activity estimation, as recommended in the literature, the acquired acceleration data from activPAL™ were exported as 15 s epochs. The number of steps per each epoch, and the number of total steps per day for 5 days (excluding the first and last days, when measurements were less than 24 h long) were computed to estimate the physical activity level of the participants (Tudor-Locke and Bassett, 2004; Tudor-Locke et al., 2011). The data were processed and analyzed using the software provided by Pal Technologies Ltd, Scotland, UK.

Force and EMG data of the knee were processed in Matlab R2017a (The MathWorks, Inc., Natick, MA, USA). Force data were filtered using a first-order, low-pass Butterworth filter at a cut-off frequency of 15 Hz. EMG signals from each muscle were band-pass filtered (20–450 Hz) using a sixth-order Butterworth filter. The root mean squared (EMG RMS) value of the EMG signal was calculated using 100 ms moving windows, and normalized with EMG RMS of the MVC trials. The first 1/3 task-period of each individual was referred to as the “baseline” period, and the force and EMG signals from this period were used for computing baseline measures of force fluctuations and motor variability.

The middle 10 s of each 15-s contraction were extracted from the force and EMG data respectively for each subject and used in the data analysis. Exertions that were extremely irregular, including twitches, jerks, or gaps during the contraction, were excluded from the analysis to avoid potential confounding influence on the result considering motor variability as the primary focus of this study. This procedure resulted in removal of 9 trials in total, representing less than 0.4% of data.

Variability was characterized using a combination of linear (coefficient of variation) and nonlinear techniques (sample entropy), to quantify the amount and structure of EMG and force variability.

1. For computing the **within-cycle coefficient of variation (CV)**, mean and standard deviation of the middle 10 s of each contraction were calculated for both force and EMG data. Within-cycle CV was estimated for a whole task-period by pooling the within-cycle CV from all cycles in that task-period: i.e., by dividing pooled within-cycle standard deviation of all cycles within a task-period by the average of the means from each cycle.
2. **Between-cycle CV** was quantified as standard deviation of the means from each cycle within a task-period relative to the mean across contractions in each period.
3. **Sample entropy (SaEn)** of each contraction was computed and averaged within each task-period using the middle ten-seconds of data (force and EMG. The algorithm of sample entropy employed in this study is listed step by step below, and was adapted from Richman an colleagues (Richman and Moorman, 2000). For a time series u of *N* points, described as {u(j):1 ≤ j ≤ N}, embedding vectors *x* with time lag τ can be formed as follows:

(1) $$\begin{array}{l} {\text{x}}_{\text{m}}\left(i\right)=\{u\left(i+k^{*}\tau \right):0\leq k\leq m-1\} \end{array}$$

In total, *N* − (*m* − 1)τ embedding vectors can be formed for {*i*|1 ≤ *i* ≤ *N* − (*m* − 1)τ}, where x_m_(*i*) is the embedding vector of m data points from *u*(*i*) to *u*(*i* + (*m* − 1)τ).

Embedding dimension (m) was determined by applying the false nearest neighbor (FNN) approach (Kennel et al., 1992). Using a random selection of contractions from all subjects in the study, a function of the percentage of FNN vs. increasing embedding dimension was created. From this, the optimal embedding dimension of *m* = 4 was computed. Based on previous literature, the time lag τ for this study was chosen to be set at a delay at which the autocorrelation function of the time series falls by as much as 1/e (Rosenstein et al., 1993). The 8th contraction of every participant was randomly selected to be used to compute time lags, and the grand average of the computed time lags of τ = 5 for the knee extension activity and τ = 2 for the handgrip exercise were used for further analysis in this study. For each embedding vector x_m_(*i*), $C_{i}^{m}\left(r\right)$ was calculated, which was defined as the probability that any vector x_m_(*j*) is within a tolerance distance (r) of x_m_(*i*).

(2) $$\begin{array}{l} C_{i}^{m}\left(r\right)=\;\frac{\left\{Number\;of\;{\text{x}}_{\text{m}}\left(j\right)\;such\;that\;d\left[{\text{x}}_{\text{m}}\left(i\right),\;{\text{x}}_{\text{m}}\left(j\right)\right]\leq r\right\}}{N-(\text{m}-1)\tau \;} \end{array}$$

Where *d*, the distance between two vectors was defined to be:

(3) $$\begin{array}{l} d\left[x\left(i\right),x\left(j\right)\right]=\text{max}\{\left|u\left(i+k^{*}\tau \right)-u\left(j+k^{*}\tau \right)\right|:\\ \;0\;\leq k\;\leq m-1,\;i\neq j\} \end{array}$$

i.e., the maximum difference of their corresponding scalar components.

The parameter r, a positive real number, referred to as the tolerance distance, has been recommended to be chosen between 0.1 and 0.25 times the standard deviation of the time series (Richman and Moorman, 2000). 0.2 times the standard deviation was used in this study, based on prior literature (Samani et al., 2015). Φ^*m*^(*r*), representing the natural logarithms of the probability of matches $C_{i}^{m}\left(r\right)$, is given by:

(4) $$\begin{array}{l} \Phi^{m}\left(r\right)=\frac{\sum_{i=1}^{N-(m-1)\tau }\ln[C_{i}^{m}\left(r\right)]}{N-(\text{m}-1)\tau } \end{array}$$

Based on the above steps, sample entropy (SaEn), showing the negative logarithm of the relationship between the probability that two sequences coincide for m+1 and m points was then computed as follows:

(5) $$\begin{array}{l} SaEn\left(m,r,N\right)=-\ln(\frac{\Phi^{m+1}\left(r\right)}{\Phi^{m}\left(r\right)}) \end{array}$$

### Statistical analysis

A two-factor analysis of variance (ANOVA) was first performed to study the effects of gender and obesity on endurance time. This was followed by a step-wise multiple linear regression model to predict endurance time, in which the “participant group” (i.e., participant obesity and gender groupings), and relevant individual-specific factors were included as predictor variables. Individual-specific factors that were explored in these models were task-related strength measured prior to the start of the protocol and force variability and muscle activation variability at baseline. Force variability was quantified as CV and SaEn and variation in muscle activation patterns were quantified by within-cycle CV, between-cycle CV, and SaEn. Two-way ANOVA models were further used to understand whether the individual predictor variables (i.e., force and muscle activation variability) showed any systematic associations with gender and obesity. JMP® was used for all statistical analysis and a significance level of *p* < 0.05 was used to accept statistical significance.

## Results

### Demographics and physical activity measurements from participants

While gender and obesity related differences were observed in anthropometry, no obesity differences were observed in strength (Table 1). Males were taller (*p* < 0.001), heavier (*p* < 0.001), exhibited greater knee extension strength (*p* < 0.001), and showed greater waist circumference (*p* < 0.001) than females. Obese participants were heavier (*p* < 0.001), had higher BMI (*p* < 0.001), and exhibited higher waist circumference (*p* < 0.001) than non-obese individuals (by design). For physical activity estimated as the average number of steps per day, according to criteria found in Tudor-Locke (2010), Tudor-Locke et al. (2011), and Tudor-Locke et al. (2016), the normal weight group were relatively low in activity level (approximately 7,500 steps/day), and the obese group were sedentary or inactive (less than 5,000 steps/day). The difference between the two groups in physical activity were statistically significant (*p* < 0.001).

### Knee extension endurance time, as a function of obesity and gender

**Figure 2:**
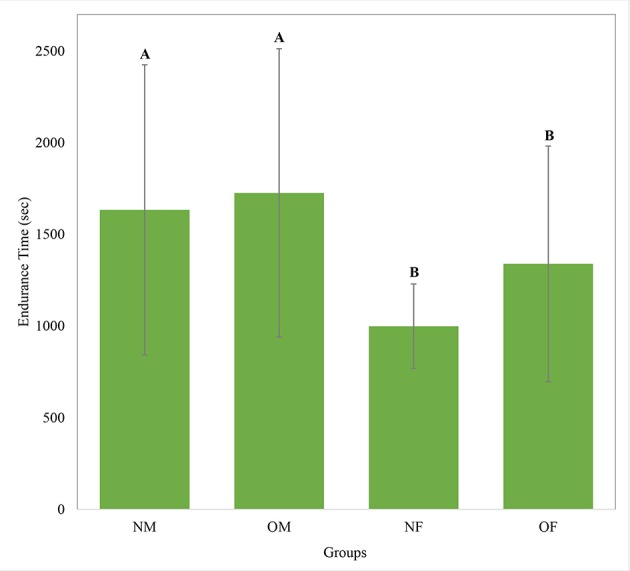
Knee extension endurance times for each group with the between-subject standard deviation shown as error bars. NM- non-obese male; OM- obese male; NF- non-obese female; OF- obese female. Means with different letters are significantly different.

Overall, the average knee extension endurance time was 1,431 (SD 700) seconds across all participants. The average endurance times in each obesity and gender group, as illustrated in Figure 2, show that male participants had 43% longer endurance time compared to females (*p* = 0.005). No statistically significant difference between obese and non-obese groups was observed (Table 2). As the Vastus Lateralis and the Rectus Femoris are both knee extensor muscle groups in the thigh having very similar functions during the knee extension task, results of these two muscles presented very similar trends. Hence, only the results of the Vastus Lateralis are considered in the following sections, considering the fact that this muscle presented the strongest trends.

**Table 2:** Main and interaction effects of obesity and gender on endurance time, along with effect sizes.

**Source**	**DF**	**Sum of squares**	**F Ratio**	***p***	**Effect size (Partial η^2^)**
Obesity	1	710.97	1.47	0.23	0.03
**Gender**	1	3972.42	8.22	0.006^*^	**0.14**
Obesity^*^Gender	1	233.89	0.48	0.49	0.007

*Statistically significant results highlighted in bold*.

### Results of multiple linear regression functions for predicting endurance time

A regression model with only participant group (coded using gender and obesity variables: i.e., NM, NF, OM, and OF as in Figure 2) as an explanatory variable and endurance time as the outcome measure was significant (*p* = 0.03) with a coefficient of determination (*R*^2^) of 16.2%. When the individual-specific variables of knee extension strength, force variability (i.e., CV and SaEn) at baseline, and muscle activation variability (i.e., within-cycle CV, between-cycle CV and SaEn of vastus lateralis) were included as explanatory variables in a multiple regression procedure, the model *R*^2^ increased to 49% (i.e., 49% of the variance in endurance time was predicted by the model) and the model was statistically significant [*F*_(9, 44)_ = 4.3; *p* = 0.0005]. Knee extension strength, force fluctuation CV at baseline, and between-cycle CV of vastus lateralis EMG were significant in the resultant model (statistical results reported in Table 3).

**Table 3:** Multiple linear regression results for predicting knee extension endurance time as a function of gender and obesity group, and individual-specific factors.

**Model terms (independent variables)**	**Endurance time (dependent variable)**		
	**Estimated coefficient**	***p***	**Effect size (partial η^2^)**
Intercept	141.25		
Group	NF: −14.44; NM: 10.53; OF: −8.21	0.07	0.15
**Knee extension strength**	−0.24	**0.02**	**0.13**
**Force CV**	−713.5	**0.03**	**0.10**
Force SaEn	−233.7	0.66	0.01
VL EMG within cycle CV	263.89	0.08	0.07
**VL EMG between cycle CV**	−21.27	**0.006**	**0.16**
VL EMG SaEn	−22.06	0.40	0.02

*NM, non-obese male; OM, obese male; NF, non-obese female; OF, obese female (statistically significant results highlighted in bold)*.

**Figure 3:**
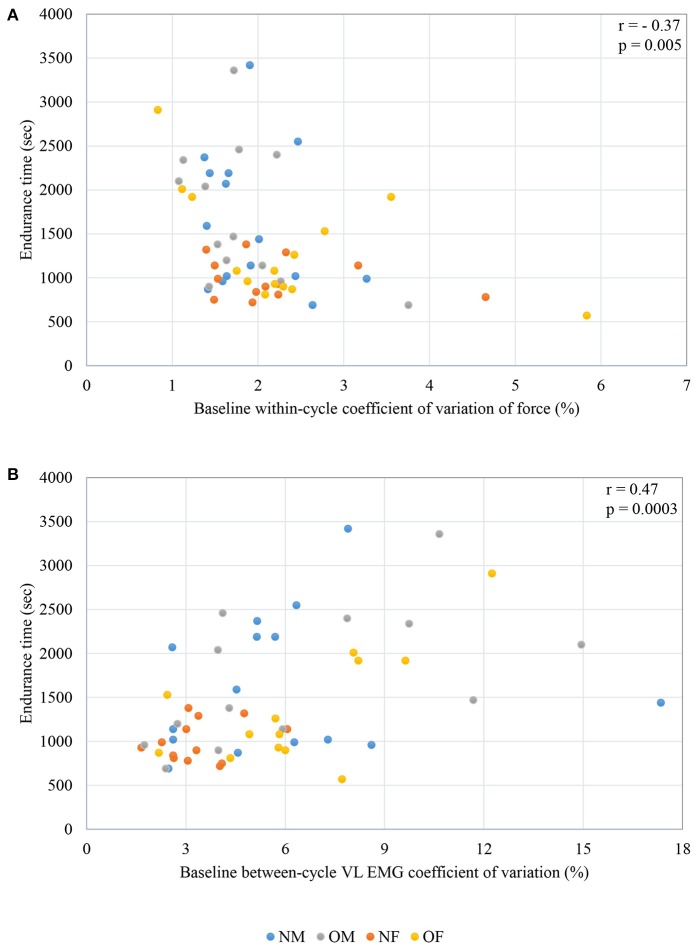
Correlations between **(A)** force CV and endurance time **(B)** between-cycle EMG CV at baseline in the Vastus Lateralis and endurance time; NM- non-obese male; OM- obese male; NF- non-obese female; OF- obese female.

When individual correlations between each of the significant predictor variables and endurance time were computed, knee extension strength did not exhibit a significant correlation with endurance time. Force fluctuation CV at baseline was negatively correlated with endurance time (*r* = −0.37, *p* = 0.005), and between-cycle CV of VL EMG was positively correlated with endurance time (*r* = 0.47, *p* = 0.0003). The individual correlations between force CV and endurance time (ET), as well as the between cycle CV of EMG with endurance time (ET) are plotted in Figures 3A,B.

### Associations of force and muscle activation variability at baseline with obesity and gender

**Figure 4:**
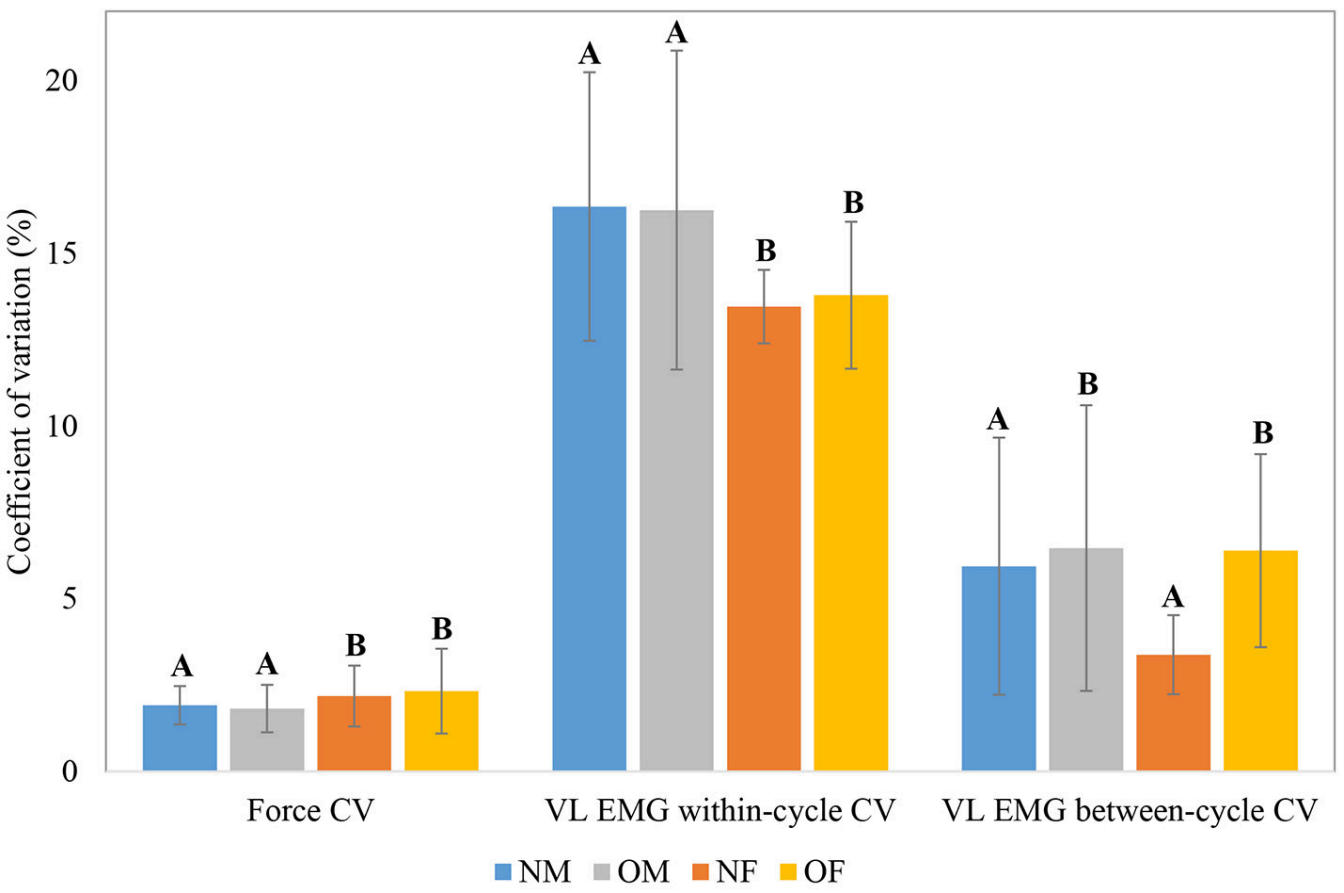
Group level means and within-group standard deviations (error bars) of force CV, VL EMG within-cycle CV and VL EMG between-cycle CV; Means with different letters are significantly different.

**Figure 5:**
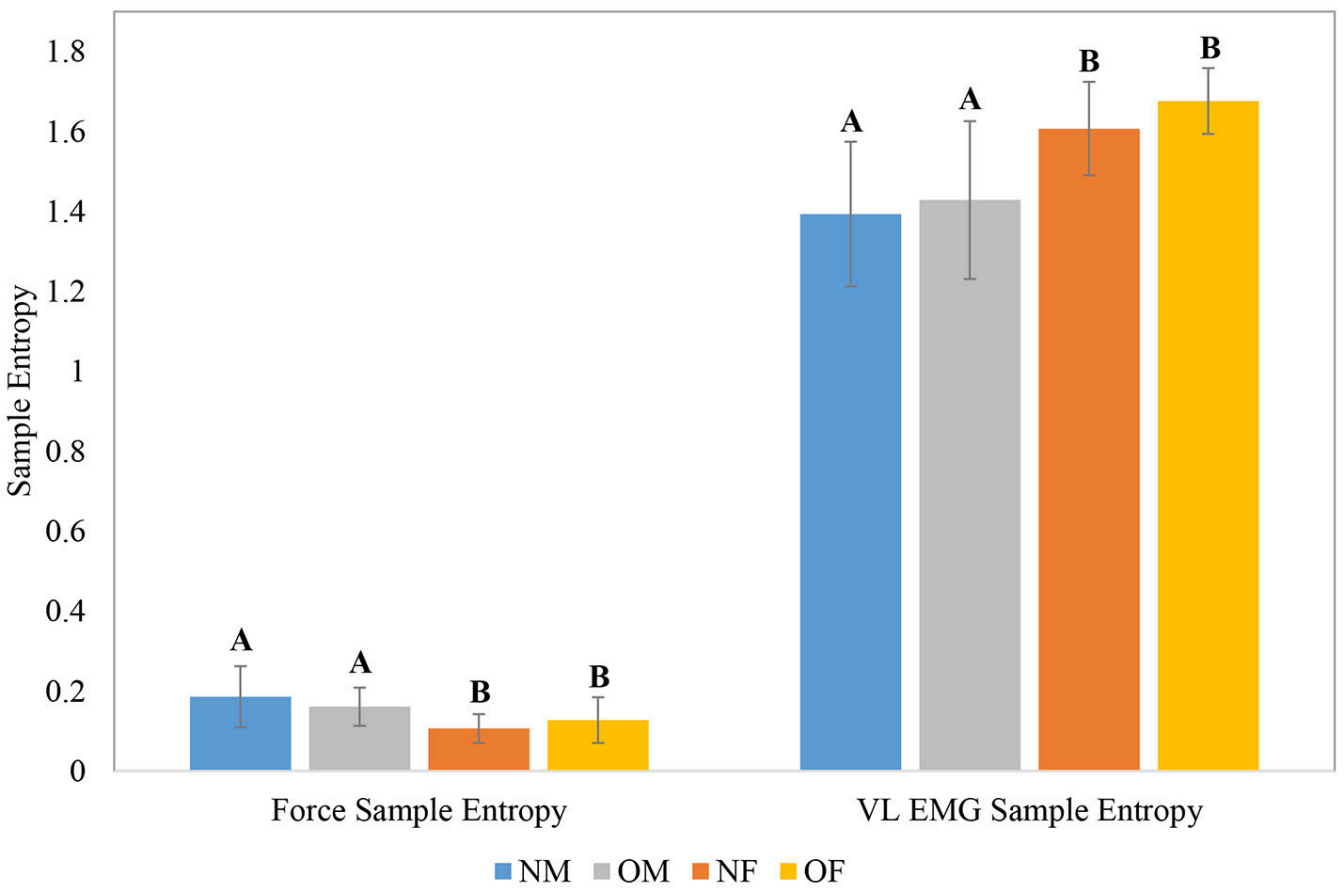
Group level means and within-group standard deviations (error bars) of force sample entropy and VL EMG sample entropy; Means with different letters are significantly different.

The means and within-group standard deviations of force and EMG CV during the baseline period of the knee extension task, as a function of participant obesity and gender are shown in Figure 4; and those of force and EMG sample entropy are shown in Figure 5. In general, males exhibited lower force CV (i.e., lower fluctuation in force control at baseline), higher force entropy, lower EMG within-cycle CV, and higher EMG sample entropy than females. The results of the statistical analysis are shown in Table 4. In terms of obesity differences, obese individuals exhibited significantly greater between-cycle CV in VL EMG than non-obese individuals. No other differences were statistically significant.

**Table 4:** Main and interaction effects of obesity and gender on force and EMG variables.

	**Force CV**		**Force SaEn**		**VL EMG within cycle CV**		**VL EMG between cycle CV**		**VL EMG SaEn**	
	***p***	**Effect size (partial η^2^)**	***p***	**Effect size (partial η^2^)**	***p***	**Effect size (partial η^2^)**	***p***	**Effect size (partial η^2^)**	***p***	**Effect size (partial η^2^)**
Obesity	0.92	< 0.001	0.9	< 0.001	0.9	< 0.001	**0.04**	**0.08**	0.21	0.03
**Gender**	**0.05**	**0.08**	**0.0006**	**0.21**	**0.0036**	**0.15**	0.14	0.04	<**0.0001**	**0.38**
Gender^*^Obesity	0.62	0.005	0.14	0.04	0.81	0.001	0.16	0.04	0.67	0.003

*Statistically significant results highlighted in bold*.

## Discussion

The present study investigated motor performance, neuromuscular control, and endurance differences with obesity and gender among older adults performing intermittent submaximal knee extension exercises. Motor performance and control were characterized by variability in force and muscle activity. Both the amount and structure of variability were quantified using the coefficient of variation (CV) and sample entropy (SaEn) metrics. In addition, variability in muscle activation within each contraction cycle as well as the cycle-to-cycle variability were quantified by computing within-cycle and between-cycle CV.

### Gender differences

Significant differences in endurance time were observed with gender, with males showing longer endurance time than females, in the knee-extension task. The majority of previous studies have reported either the same endurance time for both genders, especially at higher contraction intensities (Maughan et al., 1986), or females exhibiting better fatigue resistance than males when performing knee extensions (Clark et al., 2005; Albert et al., 2006), in contrast with what was found in the current study. Numerous other studies involving various types of exercises performed by different muscles also mostly reported a longer endurance time for female participants (Fulco et al., 1999; Hunter et al., 2004a,b). However, it is noteworthy that nearly all studies examining gender difference in fatigability focused on only young healthy adults. Our study suggests that older males particularly showed longer endurance than older females in knee extension.

Aging has been suggested to be associated with enhanced fatigue resistance, but there is a lack of research targeting the older population specifically and exploring fatigue characteristics as a function of gender. One of a few studies investigating gender and age effects on fatigability, involved a thumb adduction task and found that older adults were significantly less fatigable than young adults, and older males benefited from such age-related rise of fatigue resistance more than older females (Ditor and Hicks, 2000). The occurrence of changes in muscle fiber type distribution with aging has been demonstrated to account for the maintenance and enhancement of fatigue resistance. Older adults possess higher proportion of type I muscle fibers (slow- twitch), that help enable long endurance tasks, and fewer type II muscle fibers (fast-twitch) that are used for fast high force production but prone to fatigue. However, the amount of age-related increase in fatigue resistance can probably be modified by certain factors, such as gender. As a loss in estrogen has been seen in older females, their endurance advantage obtained from greater representation of type I muscle fibers may be mitigated, while muscular endurance of older males may predominantly benefit from histological changes alone (Ditor and Hicks, 2000). In addition to the unclear gender differences, whether the slightly greater proportion of type I muscle fiber observed in young females persists during the aging process is yet known (Hicks et al., 2001). Thus, the longer endurance time presented among males in this study could possibly result from gender differences in the age-related changes in muscle fiber distribution. There may also be other factors such as obesity, physical activity, lifestyle as well as cognitive factors, such as attitudes and beliefs about self-efficacy, that may play a role in explaining gender-related endurance differences (Weinberg et al., 1981; Cavuoto and Nussbaum, 2013a,b; Mehta and Cavuoto, 2015).

From a motor control perspective, gender differences in both force and muscle activity variability were observed, indicating that males may use a more flexible motor strategy, thus being able to prolong endurance. The force CV at baseline (inverse of force “steadiness”) was found to significantly explain inter-individual differences in endurance time, and force CV was negatively correlated with endurance time across all participants. The significant gender difference in force CV further indicated a steadier force output for males compared to females during the baseline period. We also examined force SaEn differences between genders at baseline, and force SaEn was significantly lower among females compared with males. It has been suggested that a reduction of complexity in physiological time series is associated with system dysfunction and loss of adaptability to physiological stress (Lipsitz and Goldberger, 1992). In addition, Pincus pointed out that less complexity corresponded to greater component autonomy and isolation (Pincus, 1994). As force was the product of complex interactions of neuromuscular system components, and was influenced by the pattern of activation within a muscle and across a group of synergistic and antagonistic muscles, smaller complexity in the force fluctuation may imply weaker neural compartments communication, less muscle coordination and less alterations in motor unit recruitment during static muscle contraction. Thus, a lower complexity of the force output observed among females may reflect poorer motor control and adaptation.

We interpreted the force CV and SaEn findings as jointly indicating that males controlled their force output more “tightly” than females, as reflected by the lower CV at baseline, and that they achieved this through greater corrections of the force signal, as reflected in the higher complexity of the signal as quantified by SaEn. However, we expected that such a tighter control on the force output by males would also imply that males would consequently fatigue at a faster rate, thus leading to shorter endurance times. The finding that despite more controlled force outputs, males actually showed longer endurance times than females, was unexpected and intriguing.

As for variability in muscle activations, males presented higher EMG CV in the vastus lateralis muscle (knee extensor muscle) and lower sample entropy than females. When these findings are integrated with the findings on force control and endurance time, males thus presented with higher EMG CV, lower EMG SaEn, lower force CV, higher force SaEn, and longer endurance time than females. This indicates that males somehow achieved a motor strategy in which they controlled the primary agonist muscle (vastus lateralis) to a “lower” extent, as reflected through higher CV and lower SaEn, while at the same time, achieving better force control than females. The lower SaEn observed in the extensor muscle group activation indicates that the knee extensor muscle was controlled to a lower extent among males than females, and a consequent lower neuromuscular effort by the CNS in extensor muscle control. Higher variability in muscle activation during an isometric contraction implies a more flexible motor control strategy. One possible strategy may be that alternate motor units within the same muscle, as well as other muscles with redundant function, may have been recruited and de-recruited to different extents to achieve the same overall force levels (note that the vastus lateralis is one of four knee extensor muscles in the quadriceps muscle group, indicating that this is a feasible motor control strategy). This explains both the longer endurance time that would then be achieved by not over-exerting the same motor units continually, and also the higher force SaEn as such muscle recruitment patterns would be reflected as higher complexity of the resultant force signal.

Finally, our results indicate that despite controlling a primary agonist muscle to a lower extent, males were still able to achieve better force control (lower force CV) than females. This may have been made possible by expending more effort in better antagonistic muscle control (flexor groups in this case) among males. Thus, the knee flexor muscle groups of males may obtain greater distribution of the neural effort, resulting in the lower sample entropy in the extensor muscle activity, so that the antagonists would do a better job in stabilizing the joint and assisting the control of the force output. This may have allowed the VL muscle activity to be more variable, which increased the fatigue resistance of the primary extensor, without affecting performance. Even though this hypothesis could not be verified in this study as the knee flexors were not included here, it is supported by findings from previous studies that demonstrated that women exhibited greater variation in antagonistic muscle activations and motor performance in spatial control tasks (as opposed to force control tasks here) than men (Casamento-Moran et al., 2017). From a control perspective, other studies (Granata et al., 2002) have also observed lower active stiffness of the quadriceps muscle in females during isometric knee extension when compared to males, which may in turn cause gender-based differential recruitment of agonists and antagonists in order to maintain sufficient joint stiffness.

Thus, male and female older adults differed in motor control patterns during intermittent submaximal knee extension exercises. Males seem to have employed motor strategies that better “distributed” the neural efforts across synergists and antagonists to achieve better performance, so that the primary extensors would gain more freedom to seek for less fatigable motor solutions to slow down exhaustion.

### Obesity differences

Obesity has been found to be associated with impaired functional performance such as shorter endurance, faster development of discomfort, greater fatigue, inefficient motor control, reduced relative strength, and task performance compared with normal weight people (Kankaanpää et al., 1998; Maffiuletti et al., 2007; D'Hondt et al., 2008; Eksioglu, 2011; Shortz and Mehta, 2013; Cavuoto and Nussbaum, 2014). Thus, we expected the obese group to exhibit less variable and complex muscle activity as a reflection of poorer motor control and adaptation, which would finally result in poorer force control and shorter endurance time. However, we did not observe any obesity-related differences in endurance time or force fluctuation CV in this study. Force sample entropy was lower among the obese than non-obese participants during the knee extension exercise, indicating a dysfunction and reduced effectiveness of neuromuscular control (Lipsitz and Goldberger, 1992; Pincus, 1994) within each constant contraction interval. On the muscle activation side, no difference in the within-cycle muscle variability (VL EMG CV) was observed between obese and non-obese individuals. How older obese participants achieved the same performance as the non-obese participants when bearing the physiological and motor control disadvantages described above, thus emerged as a critical question that needed to be addressed.

Obesity has been shown to be linked with poorer motor performance in terms of lower motor unit activation (as elicited by electrical stimulation) during knee extension (Blimkie et al., 1990) and ankle dorsiflexion (Pajoutan et al., 2016), for obese when compared to non-obese individuals. One study conducted by Shortz and Mehta involving intermittent handgrip and elbow flexion reported that while obesity decrements in motor performance (quantified as force fluctuation) was found during handgrip, no obesity differences were seen during elbow flexion (Shortz and Mehta, 2013). The authors explained that obesity-related declines in performance may be muscle dependent, and that obesity-related performance declines during handgrip exertions could potentially be influenced by lower neural control (Shortz and Mehta, 2013). While the above studies involved young adults, older obese adults have been shown to maintain motor performance by increasing neural control as compared to non-obese adults, as found in a gait study (Osofundiya et al., 2016), however, as tasks become more difficult (i.e., increase in effort over time), neural control may not be sufficient to offset obesity-related performance differences.

In the present study, our finding of lower complexity (SaEn) in the force signal, and no differences between obese and non-obese groups in within cycle variability or complexity of muscle activations, seem to contradict the previous speculations of increased neural control in obese older adults. However, considering that the test was an intermittent task involving regular rest periods between exertions, cycle-to-cycle muscle variability also deserved attention as it may reflect the neuromuscular control from a different perspective. It was observed that obese people displayed a significantly more variable muscle activation pattern between cycles. The increased variability across contractions among obese individuals can be interpreted as a neuro-motor strategy that they used, to prolong endurance, especially as between-cycle variability was significantly positively correlated with endurance time. As suggested in previous studies, motor variability may be a representation of the motor unit recruitment pattern (Newell and Corcos, 1993; Davids et al., 2003; Stergiou, 2004). A previous study examining the motor unit recruitment pattern in the Vastus Lateralis during submaximal intermittent knee extensions found a monotonic decrease in the recruitment threshold of all motor units, and that an increasing number of motor units were continuously active in subsequent contractions, all without a change of the recruitment order (Adam and De Luca, 2003). Thus, the reason for a varying cycle-to-cycle muscle activity could be that, during a cyclic endurance task, new motor units are progressively recruited after the activation of old motor units in subsequent contractions following the same recruitment order, due to a decrease in the recruitment threshold of the motor units. Therefore, it was reasonable to speculate that the speed of such decrease was faster among obese people, leading to a faster increase in the number of motor unit recruitment along contractions, which was reflected by the greater cycle-to-cycle variability of EMG.

Through the recruitment of more (and different) motor units than non-obese people, individual motor units of the obese participants would be able to be more “relaxed” while still maintaining the required effort output at the same time. A greater cycle-to-cycle variability in motor activation was thus reflective of a stronger neural control strategy that is likely to be beneficial in terms of decelerating fatigability among obese older adults. This finding from our study is, thus, in agreement with that from Osofundiya and colleagues that obesity was associated with higher neural costs that may contribute to comparable performance observed in obese and non-obese groups among old people (Osofundiya et al., 2016). Whether such differences in neural control strategies between the obese and non-obese groups to achieve similar endurance time is evident in neural activation patterns as observed through brain imaging studies, and what the consequences of higher neural costs of movements would be, to obese older adults in the long-term, would be significant avenues for further research.

### Limitations

There are some limiting factors that reduced the generalizability of the current study. Various methods have been used as criteria to determine obesity, such as body fat percentage, hip-to-waist ratio, body density, and BMI. Different methods may yield opposite results (e.g., some people who have high body fat percentage may also have an ideal hip-to-waist ratio). As BMI was chosen as the only approach in this study to determine obesity, the grouping, and results obtained may be biased. Another limitation was that the antagonistic muscle activities during the knee extension were not recorded, which left the coordination patterns between muscles unclear, also made the interpretation of the results and getting the full picture of neuromuscular controls more difficult. This limited our ability to completely explain our findings, thus needing further test and demonstration.

### Future directions

Submaximal isometric contractions were investigated in this study with a fixed work cycle and intensity of exertion. Future studies could continue this investigation by examining motor variability and neuromuscular control patterns under different cyclic patterns and force intensities. Moreover, as the vast majority of jobs and activities in the workplace and daily life involve dynamic motions and various levels of force exertions, future research should consider using more realistic and representative tasks to obtain more generalizable results. To gain a more complete picture of the neuromuscular control and coordination strategies used by different individuals, it is also recommended to incorporate measurements from, and activities involving agonistic, synergistic, and antagonistic muscles, to the most feasible extent.

Finally, based on our own and previous researches of others, it is clear that there is a need for a comprehensive and holistic model that brings together both basic and higher level factors to understand control and performance. We recognize that this is a very complex problem as there are multiple such factors, including personal factors (e.g., age, gender, obesity), biological factors (e.g., muscle physiological, neurological, hormonal factors), behavioral factors (e.g., motor control and coordination, strength, physical fitness), environmental, psychological, and social factors. This would require major efforts across multiple and currently-independent research disciplines but this now seems a necessary and critical requirement for major advances in this field.

## Conclusions

This study was a basic investigation on obesity- and gender-related differences in endurance among a cohort of older adults, and inter-individual differences in motor variability that may explain differences in endurance time. Males and females who were obese or non-obese, differed in the neuromuscular control mechanisms to resist fatigue and prolong endurance during intermittent submaximal muscle contractions. Males exhibited stronger fatigue resistance than females during the knee extension task, which was probably attributed to a greater antagonistic/synergistic control that slowed down the development of fatigue by allowing higher muscle variability in the primary knee extensor, while still maintaining the required force output. A motor control pattern with a greater cycle-to-cycle muscle variability at baseline was found to be associated with longer endurance time for the whole group, and the obese group exhibited significantly higher between-cycle variability than the non-obese group. We argue that this is reflective of stronger neural control exercised by the obese individuals to achieve similar endurance as non-obese older adults. Our findings enhance the theoretical understanding of the underlying neuromuscular control patterns and their relationship with fatigue for different individuals. Given that both aging and obesity rates are rising continuously and becoming a substantial health and safety priority for the society, the results from this study are both timely and critical.

## Ethics statement

This study was carried out in accordance with the recommendations of the Federal Regulations for Protection of Human Research Subjects (45 CFR 46). The protocol was approved by the Institutional Review Boards at Texas A&M University and Virginia Tech. All subjects gave written informed consent in accordance with the Declaration of Helsinki.

## Author contributions

XD was primarily responsible for analyzing the data and preparing the manuscript. JR was responsible for collecting and processing the data. RM was responsible for conceptualizing and designing the study. DS was primarily responsible for directing the data analysis, interpreting the findings, and manuscript writing. All authors contributed to critically reading and editing the manuscript content and language.

### Conflict of interest statement

The authors declare that the research was conducted in the absence of any commercial or financial relationships that could be construed as a potential conflict of interest.
